# Pilot Randomized Clinical Trial of a Passive Non-invasive Positive End-Expiratory Pressure (PEEP) Device for Delivering Positive Pressure Therapy Compared to Standard Care in Non-critically Ill Patients With COVID-19

**DOI:** 10.7759/cureus.71267

**Published:** 2024-10-11

**Authors:** Lalit Gupta, Abhinav Bassi, Bharath Kumar Tirupakuzhi Vijayaraghavan, Lovenish Bains, Kirti Nath Saxena, Naomi E Hammond, Sheila Myatra, Jigeeshu Divatia, Senthilkumar Rajagopal, Gian Luca Di Tanna, Xiaoqiu Liu, Serena Knowles, Nikita Bathla, Vivekanand Jha, Balasubramanian Venkatesh

**Affiliations:** 1 Anesthesiology and Critical Care Medicine, Maulana Azad Medical College, New Delhi, IND; 2 Public Health, The George Institute for Global Health, New Delhi, IND; 3 Critical Care Medicine, Apollo Hospitals, Chennai, IND; 4 Surgery, Maulana Azad Medical College, New Delhi, IND; 5 Surgery, Lok Nayak Hospital, New Delhi, IND; 6 Anesthesiology, Maulana Azad Medical College, New Delhi, IND; 7 Anesthesiology, Lok Nayak Hospital, New Delhi, IND; 8 Critical Care Medicine, The George Institute for Global Health, Sydney, AUS; 9 Medicine, University of New South Wales, Sydney, AUS; 10 Intensive Care Unit, Malcolm Fisher Department of Intensive Care, Royal North Shore Hospital, Sydney, AUS; 11 Anesthesiology and Critical Care Medicine, Tata Memorial Hospital, Mumbai, IND; 12 Critical Care Medicine, Tata Memorial Hospital, Mumbai, IND; 13 Health Economics and Medical Statistics, The George Institute for Global Health, Sydney, AUS; 14 Epidemiology and Biostatistics, The George Institute for Global Health, Sydney, AUS; 15 Administration, The George Institute for Global Health, New Delhi, IND; 16 Medicine, Imperial College London, London, GBR; 17 Critical Care Medicine, The George Institute for Global Health, New Delhi, IND; 18 Intensive Care Medicine, Princess Alexandra Hospital, Auchenflower, AUS; 19 Intensive Care Medicine, University of Queensland, Brisbane, AUS; 20 Intensive Care Medicine, The Wesley Hospital, Auchenflower, AUS

**Keywords:** covid-19, materialise passive non-invasive peep device mask, non-invasive peep device, respiratory support, ventilatory support

## Abstract

Background: During the COVID-19 pandemic, there were reports of a shortage of ventilators and oxygen supply, particularly in resource-limited settings. We report the preliminary evaluation of a non-invasive positive end-expiratory pressure (PEEP) mask in hospitalized non-critically ill patients with COVID-19.

Methods: We randomly assigned hospitalized adult patients with confirmed COVID-19 infection and requiring greater than 40% supplemental oxygen to either standard care oxygen delivery (control) or via Materialise passive non-invasive PEEP device mask (intervention; Belgium). The primary outcome was a change in mean respiratory rate from baseline over the first three hours after the commencement of the intervention. Secondary outcomes included dyspnea score, need for escalation of respiratory or cardiovascular support, days alive and free of ICU, and day-28 mortality.

Results: Between April 30, 2021, and October 10, 2021, we enrolled 132 (65 control, 67 intervention) patients in the study. The mean respiratory rates at baseline were 23 ± 3 and 23 ± 3 in the control and intervention groups, with no significant differences at three hours (23 ± 2.3 vs. 23 ± 2.1, p=0.14). The control group had a higher mean dyspnea score compared to the intervention group (day 5: 5.4 ± 1.6 vs. 4.7 ± 1.4, p=0.015; day 6: 4.7 ± 1.7 vs. 4.0 ± 0.7, p=0.008). A higher proportion of patients in the control group required escalation of respiratory support (38%), as compared to intervention (12%) (p=0.0004). The two groups had no significant differences across other secondary outcomes or with respect to adverse events (barotrauma, aspiration pneumonia, need for vasopressor support).

Conclusions: The use of the novel mask compared to standard care in hospitalized non-critically ill patients with COVID-19 was not associated with reductions in the respiratory rate but was associated with a reduction in the need for escalation of respiratory support without an increase in adverse effects. Large-scale clinical trials of this device are warranted.

## Introduction

Patients with severe COVID-19 infection may develop acute lung injury with hypoxemic respiratory failure [1]. A substantial proportion of these patients need supplemental oxygen. About 20% of patients hospitalized with COVID-19, particularly during the alpha and delta waves, required mechanical ventilatory support, either non-invasive or invasive, with attendant increases in mortality rates [2].

Common modes of delivering oxygen include nasal cannulas (flows up to 6 L/min) and face masks (oxygen flows 6-10 L/min). These devices deliver oxygen at low oxygen flow rates relative to the peak inspiratory flow rate. Tachypnoeic patients have high inspiratory flow rates and often entrain room air, which in turn dilutes the fraction of inspired oxygen (FiO2). The failure of oxygen masks to deliver the desired FiO2 can be improved by incorporating a reservoir bag in the mask by sealing the upper airway (nose and mouth) from the environment via a continuous positive airway pressure (CPAP) mask, thereby incorporating positive pressure, or by high-flow nasal cannula (HFNC). CPAP and HFNC devices provide positive end-expiratory pressure (PEEP) and may reduce lung injury by reducing the vigorous inspiratory effort and the associated high transpulmonary pressure swings and local lung overstretch [3,4].

HFNC units need an air/oxygen blender, an apparatus for heating and humidification, and a source of high-flow oxygen (typical flows of 30-60L/min). CPAP and non-invasive ventilation masks need an ICU ventilator or a stand-alone machine. These are not universally available in resource-limited settings and need trained healthcare workers and resources for safe delivery and the attendant concerns about aerosolization of SARS-CoV-2 [5,6]. Their use requires healthcare workers to use droplet and contact precautions with fit-tested N95 masks.

During the COVID-19 pandemic, there were reports of a shortage of ventilators [7-9] and oxygen supply [10], particularly in resource-limited settings. Biomedical scientists have developed novel devices to meet this challenge and to enable respiratory support, one of which is a non-invasive PEEP (NIP) mask. By providing a tight seal and PEEP, the NIP mask enables oxygen delivery using standard wall oxygen outlets or oxygen cylinders without the need for a mechanical ventilation system. It also obviates the need for humidification or heating devices while still delivering PEEP. Besides a lower capital cost, the ease of use of an NIP mask allows its implementation in regional/district hospitals and primary health care centers.

We report the preliminary evaluation of a NIP mask in hospitalized non-critically ill patients with COVID-19 requiring supplemental oxygen.

## Materials and methods

Methods

The study was an investigator-initiated prospective, parallel arm, open-label, phase 2b randomized controlled trial of oxygen delivery devices in non-critically ill patients hospitalized with COVID-19. The trial was conducted in two centers: Maulana Azad Medical College and Lok Nayak Hospital, New Delhi, India, and Apollo Hospitals, Chennai, India. The trial sponsor, The George Institute for Global Health, Sydney, Australia, and New Delhi, India, coordinated operational processes. The trial was approved by relevant ethics committees (The George Institute for Global Health Institutional Ethics Committee; project number: 15/2020) and was registered in April 2021 with the Clinical Trial Registry - India (CTRI registration number: CTRI/2021/04/0331750). The study was conducted in accordance with the Good Clinical Practice guidelines of the International Council for Harmonization and other national and local regulations. We report the results of the trial using the CONSORT 2010 statement: extension to randomized pilot and feasibility trials [11].

**Figure 1:**
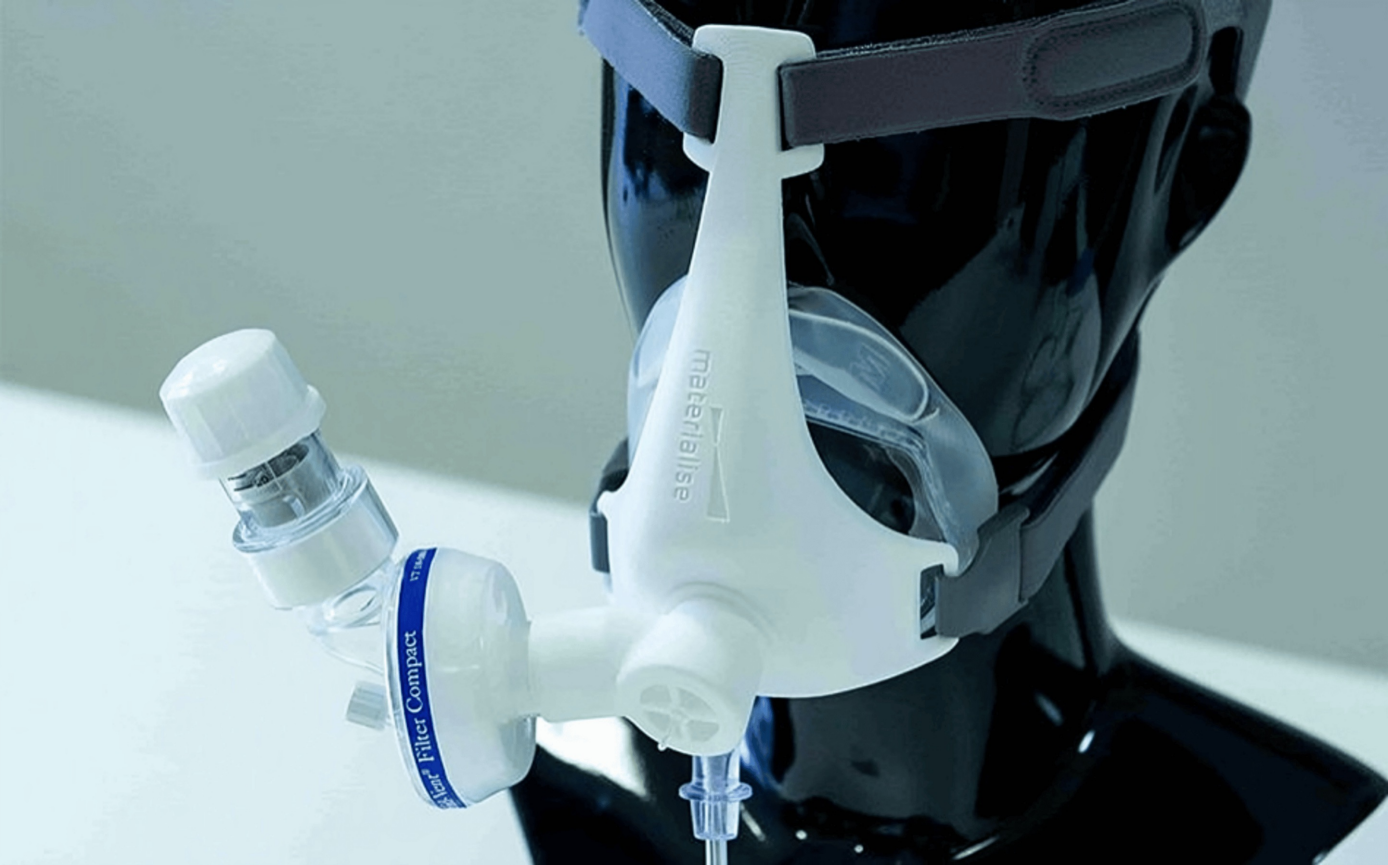
Materialise 3D-printed oxygen PEEP mask NIP mask set-up 3D: three dimensional, NIP: non-invasive positive end-expiratory pressure, PEEP: positive end-expiratory pressure

The manufacturer, Materialise, Belgium, supplied the NIP masks for the trial [12]. The NIP mask is a tight-fitting face mask, available in different sizes, and has a 3D-printed connector that attaches to a viral filter and a PEEP valve (Figure 1).

Initial clinical testing on five healthy volunteers indicates that (a) standard hospital tubing fits the O2/pressurized air inlet, (b) no indication of CO2 build-up was observed, (c) oxygen flows of 4-10 liters/min achieved the desired PEEP targets, ranging from 8-12 cm H2O, (d) the tensile strength of the Materialise Passive NIP connector was found to be sufficient, and (e) the Materialise passive NIP connector allowed for the inhalation of sufficient ambient air (personal communication with Materialise).

Study population

Patients were eligible for inclusion if they were adults (>18 years of age), hospitalized with laboratory-confirmed COVID-19 infection, and requiring greater than 40% supplemental oxygen (FiO2 >0.4 or at least 6L) via either nasal cannula or a simple face mask to maintain SpO2 greater than 93%.

Patients were excluded if they were obtunded, if the attending clinician was not committed to the patient receiving mechanical ventilatory support, or if the clinician felt that the patient was in respiratory distress with an imminent need for escalation of treatment, including intubation and mechanical ventilation. Patients were also excluded if they had an advanced health directive limiting therapy, were pregnant, were on home non-invasive ventilation, or had previously participated in the PROVE trial. If eligible, informed consent was obtained from the patient or legal surrogate before randomization.

Randomization

Randomization was conducted through a password-protected, secure website using a central, computer-based randomization program. Randomization was stratified by participating institutions and the age of participants (age below 60 years, or 60 years or above). Participants were randomized 1:1 to either standard care oxygen delivery or oxygen delivery via Materialise passive NIP mask.

Interventions

Standard Care Oxygen Delivery (Control Arm)

Standard care oxygen delivery was defined as the delivery of oxygen via either a nasal cannula (also referred to as nasal prongs) or a simple face mask (such as a Hudson mask). Oxygen delivery was titrated to a target SpO2 of 93% or greater.

Oxygen Delivery via Materialise Passive NIP Device Mask (Intervention Arm)

Oxygen was delivered via the Materialise passive NIP mask continuously throughout the day or as tolerated by the patient for a cumulative total of at least two hours per day.

The oxygen delivery via the NIP mask was continued until any of the following: the oxygen requirements were no more than 4L/min via nasal cannula to maintain target oxygen saturations or the treating clinician determined that it should stop, either because the patient had improved or had deteriorated or if there was a clinical indication for escalation of respiratory support to HFNP or CPAP or bilevel positive airway pressure (BiPAP) or invasive mechanical ventilation.

Data collection and monitoring

Data were entered into an electronic data capture system, REDCap (Vanderbilt University, Nashville, TN, USA) [13], hosted at The George Institute for Global Health, New Delhi, India, by trained research coordinators. Independent monitors and central trial staff monitored and verified the trial data, including source data verification in 100% of the participants for eligibility, consent, primary outcome, and key secondary outcomes. The source data verification and review for other variables was conducted for 10% of randomly selected participants. The verification and review of the data entered into REDcap was done through a series of remote source data verification visits.

Outcomes

The trial was designed as a pilot feasibility study; the primary outcome was a change in mean respiratory rate from baseline over the first three hours after the commencement of the intervention. Secondary outcomes included dyspnea scale score, proportion of patients requiring escalation of respiratory support, duration of mechanical ventilation, need for vasopressor or inotropic support, days alive and free of ICU, day-7 mortality, and day-28 mortality.

Safety outcomes included episodes of barotrauma defined as clinical or radiological evidence of pneumothorax or subcutaneous emphysema, episodes of suspected aspiration pneumonia, and sudden unexplained hypotension.

Sample size

For this pilot study, we based our calculations on data on respiratory rates from the ISARIC registry [14]. We determined that a population of 200 patients (100 in each group) would provide 84% power to detect an 18% reduction in respiratory rate from a median baseline respiratory rate of 23 (IQR 18-26). This calculation took into account a rate of withdrawal and loss to follow-up of 10%.

Statistical analysis

Analyses were performed by intention to treat (i.e., all patients randomized, all patients analyzed as per their intervention allocation) and on complete case records. Continuous variables were summarized by mean and standard deviation, median, first/third quartile, and min/max values, while categorical variables (including dichotomous) were described by counts over the denominator and proportions of each class. Descriptive analyses were reported by the overall cohort and by the intervention arm. Safety endpoints were assessed by the Chi-square test or Fisher’s exact test if any individual cell contained a value less than 10.

A mixed model approach was used for the primary outcome using the absolute value of RR at hours 1, 2, and 3 as the dependent variable, adjusting for baseline RR. The mixed modeling included all available observations, as fixed effect covariates, the intervention group, the respiratory baseline value, the time visit (as categorical), and the interaction between the intervention group and the time visit. Baseline values and rates at 1, 2, and 3 hours were reported, and a t-test for unequal variances was used to compare mean values at three hours.

A similar mixed modeling approach was used for all continuous secondary outcomes. The differences in the proportion of patients requiring non-invasive ventilation between the two arms were tested using Fisher’s exact test.

Interim analysis

An independent data safety and monitoring committee was appointed with a plan to conduct two interim analyses after the enrolment of 50 and 100 patients, particularly for assessment of safety. Owing to the severe nature of the second wave of the pandemic in India and consequent logistic issues, only one interim analysis was conducted after the enrolment of 100 patients.

Study termination

On October 10, 2021, after having enrolled 132 patients due to a combination of funding constraints and slowing recruitment, the Trial Steering Committee decided to stop enrolment and complete a definitive analysis.

## Results

Between April 30, 2021, and October 10, 2021, we screened 182 patients, of whom 132 (65 in the control and 67 in the intervention arm) were enrolled in the study. The baseline characteristics and the physiological and clinical parameters of the two groups are provided in Tables 1-2. There was an imbalance in the proportions of the female sex (higher in the intervention group), history of diabetes mellitus (higher in the intervention group), and history of heart failure (higher in the control group).

**Table 1:** Baseline characteristics SD: standard deviation

	Control (N=65)	Intervention (N=67)
Age (years)	47.7 (13.4)	52.8 (13.0)
Sex (female)	19 (29.2%)	31 (46.3%)
Hospitalisation to randomisation duration (days)		
Mean (SD)	5.1 (3.8)	5.1 (4.0)
Number of days with symptoms before hospital admission		
Mean (SD)	3.5 (1.9)	3.2 (2.0)
Baseline height (cm)	164.9 (5.3)	164.4 (5.6)
Baseline weight (kg)	71.5 (8.3)	67.7 (8.9)
History of chronic heart disease or heart failure	5 (7.7%)	1 (1.5%)
History of chronic pulmonary disease	6 (9.2%)	6 (9.0%)
History of chronic hypertension	20 (30.8%)	22 (32.8%)
History of diabetes mellitus	23 (35.4%)	29 (43.3%)

**Table 2:** Baseline physiological and laboratory parameters

	Control (N=65)	Intervention (N=67)
Baseline temperature	37.0 (0.3)	37.1 (0.4)
Baseline heart rate	96.9 (12.9)	94.5 (12.2)
Baseline SpO2	91.3 (2.0)	91.5 (2.0)
Baseline respiratory rate	22.5 (2.9)	22.8 (2.7)
Baseline mean arterial pressure (mmHg)	92.3 (9.1)	91.2 (9.2)
Baseline inotropes received 24 hours before randomization	0 (0%)	0 (0%)
Baseline renal replacement therapy 72 hours before randomization	0 (0%)	0 (0%)
Baseline arterial PaO2	74.9 (8.5)	72.7 (6.7)
Baseline serum creatinine	0.77 (0.2)	0.7 (0.2)
Baseline serum bilirubin	0.9 (0.3)	0.84 (0.3)

Trial and concomitant regimens

Adherence to the trial protocol was 100% in both groups, with no protocol violations. All patients randomized to the intervention arm received therapy with the NIP mask for a cumulative minimum of two hours per day, while no patients assigned to the control arm received therapy with the NIP mask.

Patients assigned to the NIP mask group received the intervention for a mean duration of 5.6 +/- 1.9 days. The mean daily duration of NIP mask use was 7.5 +/-3.1 hours (range 2.0-20.0), and the mean total duration of NIP mask therapy was 43 +/-21 hours (range 4-108 hours). The highest level of extrinsic PEEP applied through the PEEP valve on days 1-5 was 10 cm of water and 5 cm of water on days 6 and 7.

**Figure 2:**
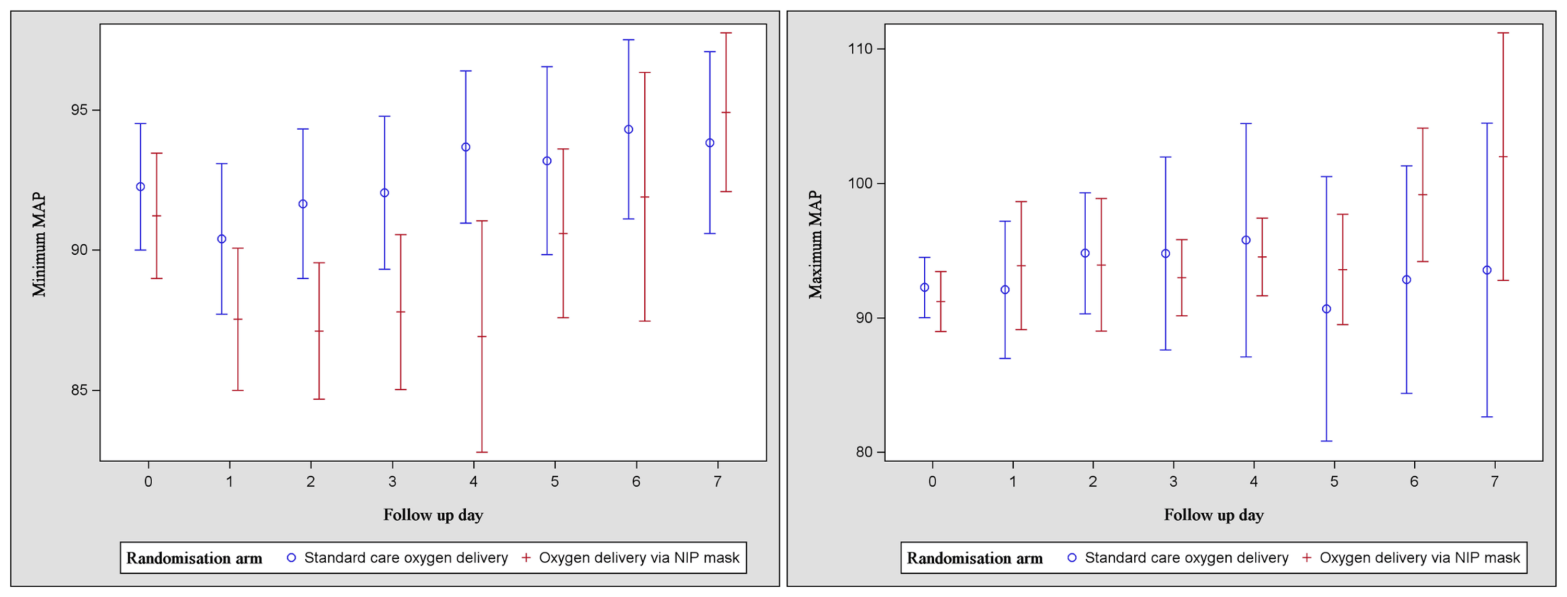
Lowest (a) and highest (b) daily mean arterial blood pressures MAP: mean arterial pressure, NIP: non-invasive positive end-expiratory pressure

**Figure 3:**
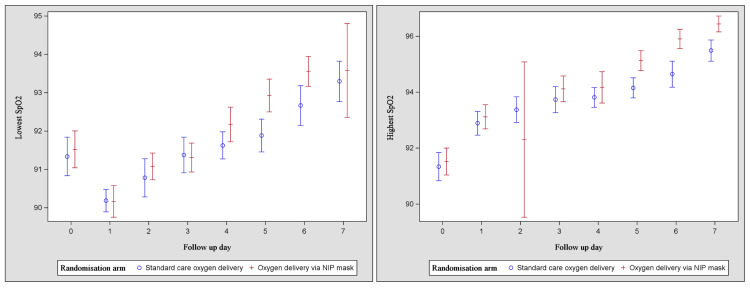
Lowest (a) and highest (b) daily oxygen saturations SpO2: oxygen saturation, NIP: non-invasive PEEP positive end-expiratory pressure

Patients assigned to the control group received the intervention for oxygen supplementation by nasal cannula or a Hudson mask for a mean duration of 5.8 +/- 1.6 days, a mean daily duration of 18.7 +/- 6.5 hours, and a mean total duration of 107.3 +/- 47 hours. The use of anti-viral, antibacterial, and immunomodulatory treatments was similar across both groups (Table 3). Between days 1 and 7, the lowest and highest mean arterial pressures and the oxygen saturations are shown in Figures 2-3.

**Table 3:** Baseline treatments SD: standard deviation, IL-6: interleukin 6

Treatment	Control (N=65)	Intervention (N=67)
Patient is receiving supplemental oxygen therapy via an open system	64 (98.6%)	65 (97.0%)
Maximum supplemental oxygen flow on an open system (within 1 hour of randomization)		
Mean (SD)	10.80 (2.5)	10.65 (2.3)
Antiviral		
Hydroxychloroquine	0 (0%)	0 (0%)
Remdisvir	28 (43.1%)	33 (49.3%)
Lopinavir	0 (0%)	0 (0%)
Ritonavir	0 (0%)	0 (0%)
Convalescent plasma	0 (0%)	1 (1.5%)
Antibacterial	58 (89.2%)	65 (97.0%)
Anti-inflammatory		
Corticosteroids	48 (73.8%)	56 (83.6%)
IL-6 inhibitors	7 (10.8%)	9 (13.4%)

Primary outcome

There were no missing data for the primary outcome. The mean respiratory rates at baseline were 23 +/- 3 and 23 +/- 3 in both the control and intervention groups. At three hours, the two groups had no significant differences in the mean respiratory rates (23 +/- 2.3 and 23 +/- 2.1, p=0.14). There were also no significant differences between the groups with respect to the change of respiratory rate from baseline, with mean differences -0.20 (95% CI -0.09 to 0.48, p=0.15).

**Figure 4:**
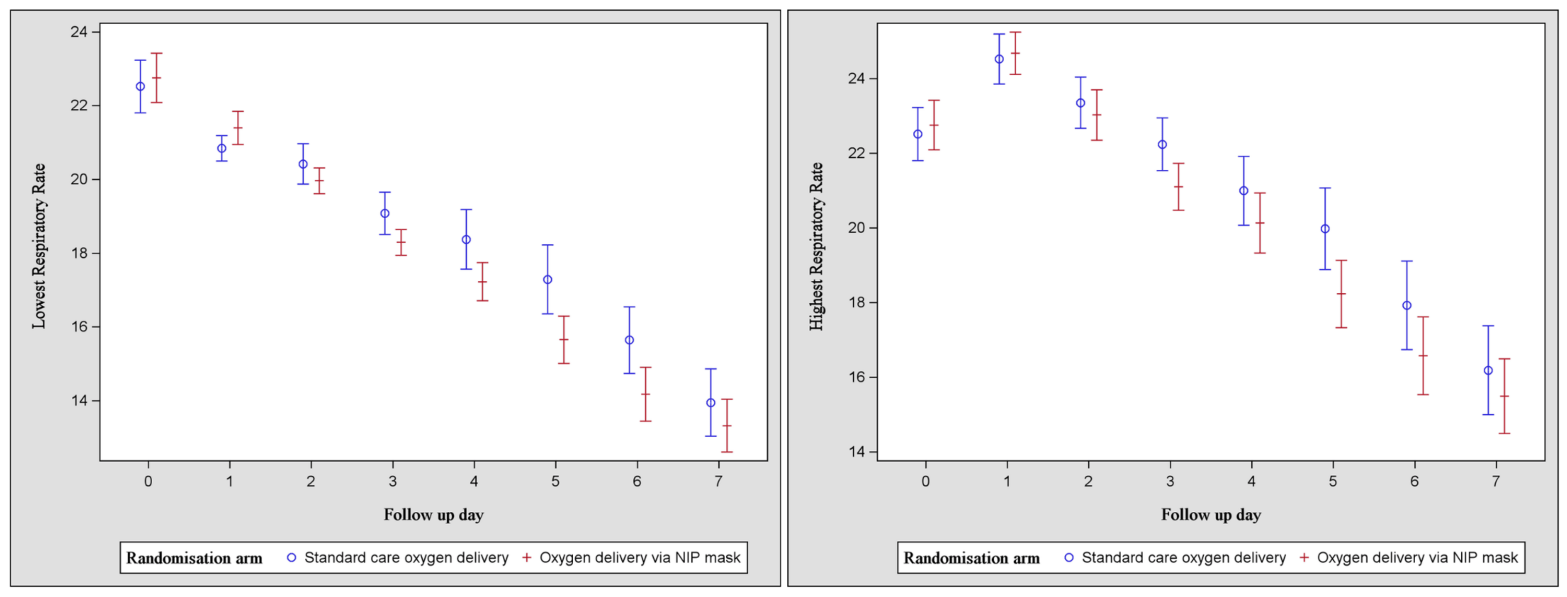
Lowest (a) and highest (b) respiratory rates between days 1 and 7 NIP: non-invasive positive end-expiratory pressure

The trends in respiratory rates in the two groups between days 1 and 7 are illustrated in Figure 4. There were statistically significant differences in favor of the intervention group with respect to the highest and the lowest respiratory rate on day 5.

Secondary outcomes

On days 5 and 6, there was a higher mean dyspnea score in the control group compared to the interventional group (day 5: 5.38 (1.608) vs. 4.65 (1.430), p=0.015; day 6: 4.70 (1.670) vs. 4.00 (0.714), p=0.008), respectively. The dyspnea scores across the seven days for the two groups are shown in Table 4. There was a higher proportion of patients requiring escalation of respiratory support to either HFNC or BIPAP in the control group (25/65, 38%), as compared to intervention (8/67, 12%) (p=0.0004). The two groups had no significant differences across all the other secondary outcomes (Table 5).

**Table 4:** Dyspnea rating

	Control (N=65)	Intervention (N=67)	T-test, p-value
Dyspnoea rating			
Day 1	5.9 (1.3)	5.9 (1.1)	0.74
Day 2	5.7 (1.3)	5.7 (1.2)	0.92
Day 3	5.5 (1.4)	5.4 (1.3)	0.74
Day 4	5.5 (1.4)	5.2 (1.3)	0.16
Day 5	5.4 (1.6)	4.7 (1.4)	0.0015
Day 6	4.7 (1.7)	4.0 (0.7)	0.008
Day 7	4.2 (1.7)	3.9 (0.8)	0.23

**Table 5:** Secondary outcomes SD: standard deviation, ICU: intensive care unit, HFNP: high-flow nasal cannula, BiPAP: bilevel positive airway pressure

Treatment	Control (N=65)	Intervention (N=67)	p-value
Hospital duration (days)			
Mean (SD)	17.3 (6.3)	16.3 (6.0)	0.37
ICU duration			
Mean (SD)	10.8 (2.8)	12.2 (10.5)	0.35
Hospital-free days			
Mean (SD)	11.1 (5.0)	12.0 (5.1)	0.31
ICU-free days			
Mean (SD)	17.2 (2.8)	17.1 (4.4)	0.83
Escalation of resp support (HFNP or BiPAP)	25 (38.0%)	8 (12.0%)	0.0004
HFNP	12 (48.0%)	1 (12.5%)	-
BiPAP	13 (52.0%)	7 (87.5%)	-
Non-invasive ventilation duration			
Mean (SD)	8.84 (12.2)	7.25 (2.8)	0.72

Adverse events

There were no significant differences between the two groups with respect to day 28 mortality, episodes of barotrauma or aspiration pneumonia, or the need for vasopressor support (Table 6). Mask discomfort was reported in 40% of patients assigned to the NIP mask.

**Table 6:** Adverse events

Treatment	Control (N=65)	Intervention (N=67)	p-value
Death up to 28 days	1 (1.6%)	0 (0.0%)	0.30
Instances of barotrauma from day 1 to day 7	0 (0%)	0 (0%)	-
Instance of aspiration-related injury from day 1 to day 7	0 (0%)	0 (0%)	-
Instance of inotrope or vasopressor use from day 1 to day 7	1 (1.6%)	3 (4.5%)	0.33

## Discussion

In this pilot feasibility trial, we found that using an NIP mask was not associated with a reduction in the respiratory rate in the first three hours after randomization compared to standard care. In patients assigned to the NIP mask, we observed improved oxygen saturations on Days 5 and 6. Fewer patients assigned to the NIP mask required escalation of respiratory support to HFNC or BIPAP. There were no differences between the two groups with respect to adverse effects.

Our pragmatic pilot feasibility trial was designed with statistical power to detect a clinically plausible effect on respiratory rate in patients with COVID-19 requiring supplemental oxygen and provide data on key secondary outcomes and safety. It was also designed to inform sample size calculations for a future phase 3 trial. For this feasibility trial, we chose respiratory rate as the primary outcome, as an elevation in respiratory rate is one of the consistently observed early signs of deterioration. To reduce bias, we used a central randomization process. We published our statistical analysis plan before the database lock and had no missing data for the primary outcome.

This represents the first evaluation of this novel mask in patients with hospitalized non-critically ill patients with COVID-19. Preliminary data indicate ease of use and safety with no increase in adverse effects compared to standard care. The longest daily duration of use was 20 hours. The design aspects of this mask lend themselves to easy use in resource-limited settings. To our knowledge, no other similar devices have been evaluated in patients with COVID-19, although trials of novel CPAP devices are currently underway for patients with respiratory failure [15]. Although this study was confined to patients with COVID-19, this device may also be useful in patients with other causes of respiratory failures, such as pulmonary edema, and in patients requiring perioperative respiratory support.

Our study had limitations. About 95% of the patients were enrolled in one center, limiting the generalisability of study results. The intervention could not be blinded, and the decision to escalate the intensity of respiratory support was left to the treating clinician. Owing to the challenges imposed by the pandemic and isolation of patients, we were unable to measure PEEP values generated in the mask during the course of the study, although the extrinsic PEEP did not exceed 10 cm of water and there were no instances of barotrauma. The study was terminated prematurely due to operational and funding constraints.

## Conclusions

In this pilot feasibility study, the use of the novel mask compared to standard care in hospitalized non-critically ill patients with COVID-19 was not associated with reductions in the respiratory rate but was associated with a reduction in the need for escalation of respiratory support without an increase in adverse effects. Large-scale clinical trials of this device are warranted, as the development of such systems would have important implications for clinical practice and cost efficiency, particularly in resource-limited settings. The efficacy of this device will need to be tested in a phase 3 clinical trial.
